# MiR-144-3p-mediated dysregulation of EIF4G2 contributes to the development of hepatocellular carcinoma through the ERK pathway

**DOI:** 10.1186/s13046-021-01853-6

**Published:** 2021-02-01

**Authors:** Shuangshuang Li, Jiajia Shao, Guohua Lou, Chao Wu, Yanning Liu, Min Zheng

**Affiliations:** State Key Laboratory for Diagnosis and Treatment of Infectious Diseases, National Clinical Research Center for Infectious Diseases, Collaborative Innovation Center for Diagnosis and Treatment of Infectious Diseases, The First Affiliated Hospital, Zhejiang University School of Medicine, 79# Qingchun Road, Hangzhou, 310003 China

**Keywords:** EIF4G2, HCC, miR-144-3p, ERK1/2, Prognosis

## Abstract

**Background:**

Hepatocellular carcinoma (HCC) is one of the most common cancers with high incidence and mortality. However, the underlying mechanisms of HCC still remain unclear. Eukaryotic translation initiation factors (eIFs) have a substantial effect on tumor development. In this study, we were aimed to investigate the role of eukaryotic translation initiation factor 4 gamma 2 (EIF4G2) in HCC.

**Methods:**

Western blot (WB) of 30 paired HCC tissues and tissue microarrays (TMAs) conducted by immunohistochemistry (IHC) in 89 paired HCC samples were performed to assess EIF4G2 expression. Clone formation, real-time cell analysis (RTCA), wound healing and transwell assays were adopted to evaluate the role of EIF4G2 on HCC cell proliferation, migration and invasion abilities. The function of EIF4G2 in HCC tumor growth was assessed in a xenograft nude mouse model in vivo. The regulation of EIF4G2 by miR-144-3p was performed by luciferase reporter assay and WB.

**Results:**

The EIF4G2 protein was clearly upregulated in HCC tissues, and high EIF4G2 expression was closely related to HCC prognosis. EIF4G2 silencing could inhibit HCC cell growth and metastasis in vitro, and suppress tumorigenesis in vivo by repressing the ERK signaling pathway. The results of luciferase reporter assays, WB and IHC staining verified that EIF4G2 was negatively regulated by miR-144. And re-expression of EIF4G2 could partially reverse the inhibiting effect of miR-144 in HCC.

**Conclusion:**

In summary, our study revealed the role of EIF4G2 in HCC development via the activation of the ERK pathway. We also found that EIF4G2 could be negatively regulated by the tumor suppressor miR-144. Our investigations indicated that EIF4G2 might be a promising therapeutic target in HCC.

**Supplementary Information:**

The online version contains supplementary material available at 10.1186/s13046-021-01853-6.

## Background

Liver cancer ranks the sixth in incidence and fourth in mortality rate among all cancers [1]. Although an increasing number of novel therapies for hepatocellular carcinoma (HCC) have been developed, such as transcatheter arterial chemoembolization (TACE) and liver transplantation, the prognosis of HCC patients is still poor, especially that of advanced HCC patients [2]. Therefore, it is very urgent to find new and effective clinical targets of HCC.

Currently, the role of eukaryotic translation initiation factors (eIFs) in cancer development has attracted considerable attention. Recent studies revealed that dysregulation of eIFs plays a major role in carcinogenesis by causing aberrant gene expression [3–5]. Here, we investigated the role of eukaryotic translation initiation factor 4 gamma 2 (EIF4G2), a member of the eIFs family, which is a translational activator during cellular stress [6–8]. Previous studies have indicated that abnormal expression of EIF4G2 plays key roles on the progression of many cancers. For example, suppression of EIF4G2 could significantly suppress the development of acute myeloid leukemia [9], diffuse large B cell lymphoma [10] and human osteosarcoma [11]. However, the role of EIF4G2 in HCC has not been reported. The data from the Human Protein Atlas database indicate that overexpression of EIF4G2 protein has a poorer prognosis in HCC [12]. The mRNA level of EIF4G2 is not significantly different in HCC and adjacent noncancer tissues [13]. Hence, we hypothesize that there is a posttranscriptional regulation of EIF4G2.

The posttranscriptional regulation mechanisms of microRNAs (miRNAs) are very prevalent [14]. Many miRNAs were found to have a large effect on HCC development. Our previous study discovered that miR-199a improved HCC chemosensitivity by targeting mTOR [15]. Here, we hypothesized that the role of EIF4G2 in HCC is regulated by miRNAs.

In this study, we found that EIF4G2 was a new disadvantageous factor in HCC. Down-regulation of EIF4G2 could inhibit HCC development through the suppression of the ERK signaling pathway. Mechanistic studies verified that EIF4G2 was negatively regulated by miR-144-3p.

## Methods

### HCC patients

Thirty paired HCC cancerous tissues and matched para-cancerous tissues were collected from patients of the First Affiliated Hospital, Zhejiang University School of Medicine, who underwent surgery between September 2016 and March 2017. The collected samples were all from patients who had histologically diagnosed as primary hepatocellular carcinoma and had not received prior treatments, such as TACE or chemotherapy before collection. The exclusion criteria were as follows: patients with other cancers, metastatic liver cancer (secondary liver cancer), previous liver transplantation, chemotherapy or TACE treatment. The research was permitted by the Medical Ethics Committee of the hospital. All tissues were immediately frozen at − 80 °C. All patients’ information is listed in Table S1.

### Cell lines and cell culture

Huh7, Huh7-Luc (Huh7 cells stably expressing luciferase), Hep3B and 293 T cells were cultured with Dulbecco’s modified Eagle’s medium (DMEM; HyCloneTM #AE29431636) and 10% fetal bovine serum (FBS; CORNING #35081006). The cell lines were harvested via trypsinization and washed with phosphate buffered saline. In addition, all cells were incubated in 5% CO_2_ at 37 °C. The Huh7-Luc cell line was constructed in our lab in a previous study [16].

### Real-time cell analysis (RTCA)

Cell proliferation assays were monitored by the *xCELLigence* Real-Time Cell Analysis S16 instrument and E-Plate 16 (ACEA Biosciences, Inc.) according to the manufacturers’ instructions. The electronic impedance of the system changed according to cell status, and cell growth was measured by using a parameter termed the cell index (CI). Fifty microliters of complete media were added to the plates, and the baseline was measured. Then, 5 × 10^3^ cells were seeded into each well and grown for approximately 80 h at 37 °C in 200 μL of culture media. The impedance was detected every 15 min after seeding. Data were analyzed by xCELLigence software, and the cell index was normalized to the data recorded at the time of cell treatment.

### RT-qPCR

RNA from cells and tissues was extracted by TRIzol (Takara #9109) according to the manufacturer’s protocol. Then, reverse transcription (RT) was performed using the PrimeScriptTM RT reagent kit with gDNA eraser (Takara #RR047A) according to the protocol. Quantitative real-time polymerase chain reaction (RT-qPCR) was conducted using TB GreenTM Premix Ex TaqTM (Tli RNaseH Plus) (Takara #RR420A). The results were quantified using the 2^−ΔΔCt^ method. In the present study, GAPDH and U6 were used as reference genes. The primers of miR-144-3p and U6 were purchased from RiboBio Co., Ltd., China. The primer sequences used are listed in Table S2.

### Matrigel invasion assay

Cell invasion ability was assayed by 8 mm Corning transwell insert chambers (Corning), which were coated with 50 μL of Matrigel® (Corning®) and placed into 24-well plates. Then the 24-well plates were placed for 30 min at 4 °C, and incubated for 1–2 h at 37 °C. A total of 1 × 10^5^ cells per well in 200 μL of serum-free culture medium was seeded into chambers after 48 h of transfection, and 600 μL of DMEM with 10% FBS was inserted into the lower chamber. After 48 h, invaded cells were fixed with 4% paraformaldehyde (Servicebio #1101) for 30 min and stained with 0.1% crystal violet (Dawen Biotec) for 30 min. After removing cells on the upper surface, the invaded cells were imaged and counted.

### Migration assay

Cell migration ability was assessed with wound-healing assays. Hep3B cells (3× 10^5^) and Huh7 cells (4 × 10^5^) after 48 h of treatment were seeded into 24 well plates. After cells reached complete confluence, linear wounds were made with 200 μL plastic pipette tips. And cells were cultured in DMEM with 1% FBS. Images were captured at 0, 12, 24, and 48 h. Three representative visual fields were selected from each dish, and the cell migration distances were measured with a microscope.

### Colony formation assay

Five hundred cells after transfection were seeded into 6 cm petri dishes with complete medium, and grown up to until visible colonies formed about 2 weeks. Cells were fixed with 4% paraformaldehyde for 30 min and then stained with Coomassie Brilliant Blue (FUDE Biological #FD0022) for 30 min. After washing with deionized distilled water, the dishes were air dried at room temperature. Colonies were counted and photographed with a Molecular Imager® Gel Doc™ XR+ imaging system (BIO-RAD). All assays were repeated three times.

### Bioinformatics analysis

The relationship of EIF4G2 and HCC prognosis was obtained from the Human Protein Atlas database. The EIF4G2 mRNA level data were obtained from the GEPIA database. miRNAs that can modulate EIF4G2 were predicted by the TargetScan, miRCODE, and starBase websites.

### Lentivirus transduction

MiR-144 overexpression and control(ctrl) lentivirus were purchased from GeneChem. Cells were seeded into 6-well plates at approximately 40–50% confluence, and 10 μL of lentivirus was added per well. 2 μg/mL puromycin (MCE #NSC3056) was added to select infected cells after 48 h of transduction. Subsequently, cells were cultured under 1 μg/mL puromycin to maintain stable infection.

### SiRNA and vector infection

Si-RNAs of EIF4G2 and a negative control RNA were purchased from GenePharma. Cells were transfected with 50 nM siRNA for 48 h at 37 °C using the Lipofectamine® RNAiMAX (Invitrogen #13778–015) based on the manufacturer’s protocol. Overexpression vectors of EIF4G2 and miR-144 and corresponding ctrl vectors were designed and synthetized by GeneChem. Transfection of each vector was carried out using Lipofectamine®3000 (Invitrogen #L3000–015). U0126 (MCE #HY-12031) was purchased from MCE. In addition, 20 μM 0126 was used to inhibit the ERK pathway. Total RNA and protein were extracted after 48 h of treatment. The transfection efficiency was verified by RT-qPCR and Western blot. Sequences of the siRNAs are listed in Table S3.

### Luciferase assay

The wild-type (WT) and mutant (MUT) luciferase reporter vectors of the EIF4G2 3′-UTR were obtained from GeneChem. A total of 1 × 10^4^ 293 T cells were seeded into 96-well plates, and then the WT and MUT reporter vectors and miR-144 overexpression vector were cotransfected with Lipofectamine 3000 into 293 T cells. Forty-eight hours later, the firefly and Renilla luciferase activities were assessed by a GloMax microplate luminometer (Promega, USA) using a Dual-Glo® Luciferase Assay System kit (E2920, Promega, USA). Renilla luciferase activities were used to evaluate transfection efficiency.

### Western blot analysis

Protein was extracted from cells and tissues with RIPA lysis buffer (Applygen #C1053), and the protein concentration was measured with a BCA assay (Thermo Fisher, #23227). Protein was separated by sodium dodecyl sulfate-polyacrylamide gel electrophoresis (GenScript #M00657), transferred from the gel to 0.2 μm polyvinyl difluoride membrane and blocked in TBS-T containing 5% bovine serum albumin (Haoke HK5021) or skim milk powder for 2 h. Then, the membrane was incubated with specific primary antibodies at 4 °C for approximately 14 h and incubated with secondary antibodies for 1 h at room temperature. The specific primary antibodies used in this paper were as follows: EIF4G2 (1:1000, CST #2182), p-ERK1/2 (1:1000, CST #4370), ERK1/2 (1:1000, CST #4695), p-p38 (1:1000, CST #4511), p38 (1:1000, CST #8690), p-JNK (1:1000, CST #9255), JNK (1:1000, CST #9252), and GAPDH (11,000, CST #5174).

### Animal studies

BALB/cJGpt-Foxn1nu/Gpt (BALB/c) female mice (4 weeks old, 12–14 g) were purchased from GemPharmatech Co., Ltd. (Nanjing, China), and housed in temperature-controlled and pathogen-free rooms. Water and food were provided freely. Huh7-Luc cells (1 × 10^6^) that had been transfected with si-EIF4G2, si-NC (negative control), miR-144 overexpression or ctrl lentivirus were injected subcutaneously into the backs of mice near their hind legs (7 mice pergroup). The left area was the ctrl treatment, and the right area was the experimental treatment. Tumor size was measured every 2 days, and tumor volume was calculated using this formula: volume = (length × width^2^)/2. Mice were sacrificed after approximately 20 days by cervical dislocation. The tumors were removed and weighed and photographed. All protocols of animal studies were approved by the Institutional Animal Care and Use Committee of the First Affiliated Hospital, Zhejiang University School of Medicine.

### Tissue microarrays and immunohistochemistry

The HCC tissue microarray chip, including total 89 pairs of HCC tumor tissues and matched para-carcinoma tissues, was purchased from Shanghai Biochip Company Ltd. IHC staining was performed to assess EIF4G2 expression in HCC tissues according to the manufacturer’s protocol using anti-EIF4G2 antibody (dilution 1:400, Abcam #ab97302). Each HCC-TMA score was analyzed based on the product of staining intensity and the percentage of positively stained cells. The staining intensity was assessed on a scale of 0–3. In this study, the positive rate was 100%. If a score was less than or equal to 1, we categorized it as the EIF4G2 low expression group, and if a score was greater than 1, we categorized it as the EIF4G2 high expression group.

### RNA profiling and RNA-seq analysis

Total RNA was isolated and purified by TRIzol reagent following the protocal, and then 2 × 150 bp paired-end sequencing (PE150) was performed on an Illumina Novaseq™ 6000. The differentially expressed mRNAs were selected with fold change > 2 or fold change < 0.5 and *p* value < 0.05, and then GO enrichment and KEGG enrichment analyses were performed on the differentially expressed mRNAs.

### Statistical analysis

Statistical analyses were performed with SPSS 18.0 and GraphPad Prism 8 software. Data were presented as the mean ± standard deviation (SD). In the HCC-TMA chip, chi-square tests were used to analyze EIF4G2 expression in HCC and matched tissues. Overall survival (OS) and disease-free survival (DFS) were assessed using the Kaplan–Meier method with the log-rank test. The correlation between the expression of EIF4G2 and PDL1 was analyzed by the Spearman’s rank correlation test. The correlation between the expression of miR-144 and EIF4G2 protein was analyzed by the Pearson’s test. The expression of miR-144 and EIF4G2 in 30 paired HCC tissues was performed using a paired t test. HRs and 95% confidence intervals (CIs) for clinical correlative factors were investigated using the Cox regression models in univariate and multivariate analyses. A value of *p* < 0.05 was considered to be statistically significant.

## Results

### EIF4G2 is overexpressed in HCC, and high EIF4G2 expression indicates poor prognosis of HCC patients

**Fig. 1:**
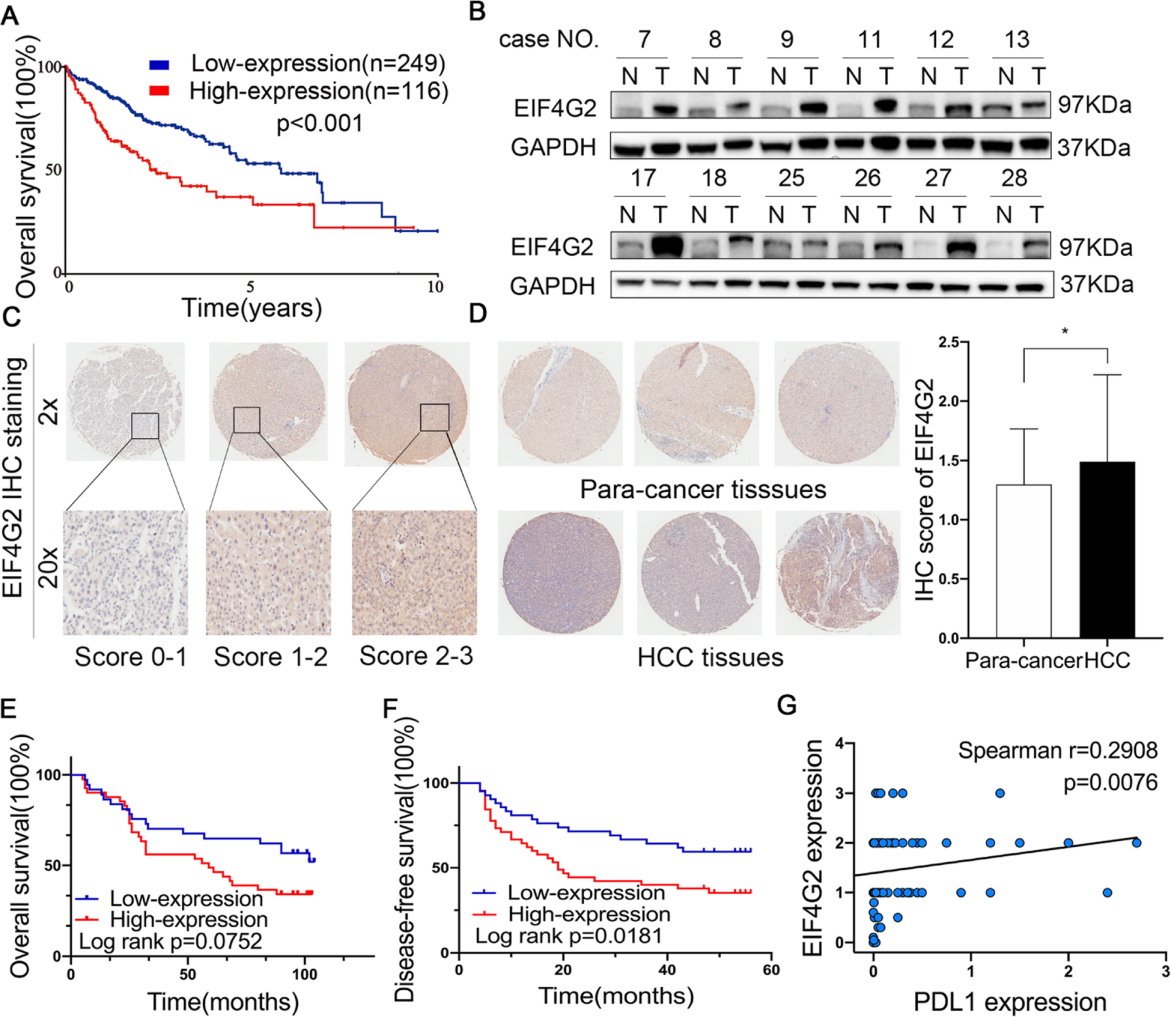
EIF4G2 is overexpressed in HCC and high EIF4G2 expression indicates poor prognosis of HCC patients. **a** OS analysis in HCC patients with high- or low-expression of EIF4G2 from the Human Protein Atlas database. **b** Representative images of EIF4G2 expression in 30 paired HCC tissues(T) and adjacent para-carcinoma tissues(*N*). **c** Representative images of EIF4G2 IHC staining with different staining scores. Images were presented at 2x magnification (up panel) and 20x magnification (lower panel). **d** Representative images and analysis of EIF4G2 IHC staining in HCC tissues and adjacent para-carcinoma tissues, images were presented at 2x magnification. **e** Kaplan–Meier curves analysis of OS in HCC-TMA patients with high- or low-expression of EIF4G2. **f** Kaplan–Meier curves demonstrating DFS in HCC-TMA patients with high- or low-expression of EIF4G2. **g** Spearman’s correlation analysis of EIF4G2 expression and PDL1 expression. *p < 0.05

As shown in Fig. 1a, the Human Protein Atlas database demonstrated that HCC patients with high levels of EIF4G2 had significantly shortened survival. To explore the role of EIF4G2 in HCC, we detected the expression of EIF4G2 in fresh frozen HCC clinical samples. The Western blot results showed that the level of EIF4G2 was remarkably upregulated in HCC tissues compared with corresponding para-carcinoma tissues (*n* = 30) (Fig. 1b and Fig. S1A-B). IHC staining was performed with an HCC-TMA chip including 89 paired HCC samples. Representative IHC staining intensity of EIF4G2 was shown in Fig. 1c, and the score ranged from 0 to 3. Consistently, the staining results also showed higher expression of EIF4G2 in HCC tumor tissues (Fig. 1d). Further analysis of associations between EIF4G2 and clinicopathological characteristics revealed how EIF4G2 overexpression affected HCC development. As shown in Table 1, EIF4G2 overexpression was significantly related to cases with a higher T stage (T2/T3, *p* = 0.01), a higher TNM stage (II/III, *p* = 0.01), HCC recurrence (*p* = 0.044) and PDL1 positive expression (*p* = 0.035). All HCC patients were followed up for at least 7 years, and compared to those with low EIF4G2 expression, the cases with higher EIF4G2 expression showed no significant difference in OS but showed a shorter DFS (*p* = 0.025) (Fig. 1e-f). In addition, EIF4G2 was positively correlated with PDL1 expression (*p* = 0.009) (Fig. 1g). Taken together, EIF4G2 might be an unfavorable prognostic marker in HCC.

**Table 1:** Correlation between EIF4G2 expression and clinicopathological characteristics

Variables	EIF4G2 expression		χ2	***p***-value
	high	low		
Age (year)			4.066	0.044
≤ 50	24	15		
>50	20	30		
Sex			0.001	0.970
Female	5	5		
Male	39	40		
Grade			3.236	0.072
1/2	25	17		
3	19	28		
T stage			6.612	**0.010**
T1	34	23		
T2/T3	10	22		
TNM stage			6.612	**0.010**
Ι	34	23		
II/III	10	22		
PDL1			4.443	**0.035**
Negative	30	20		
Positive	12	21		
Recurrence			4.048	**0.044**
No	25	16		
Yes	19	29		

Note: Bold *p* values display statistical significance, *p* < 0.05

### EIF4G2 facilitates HCC growth and metastasis in vitro

**Fig. 2:**
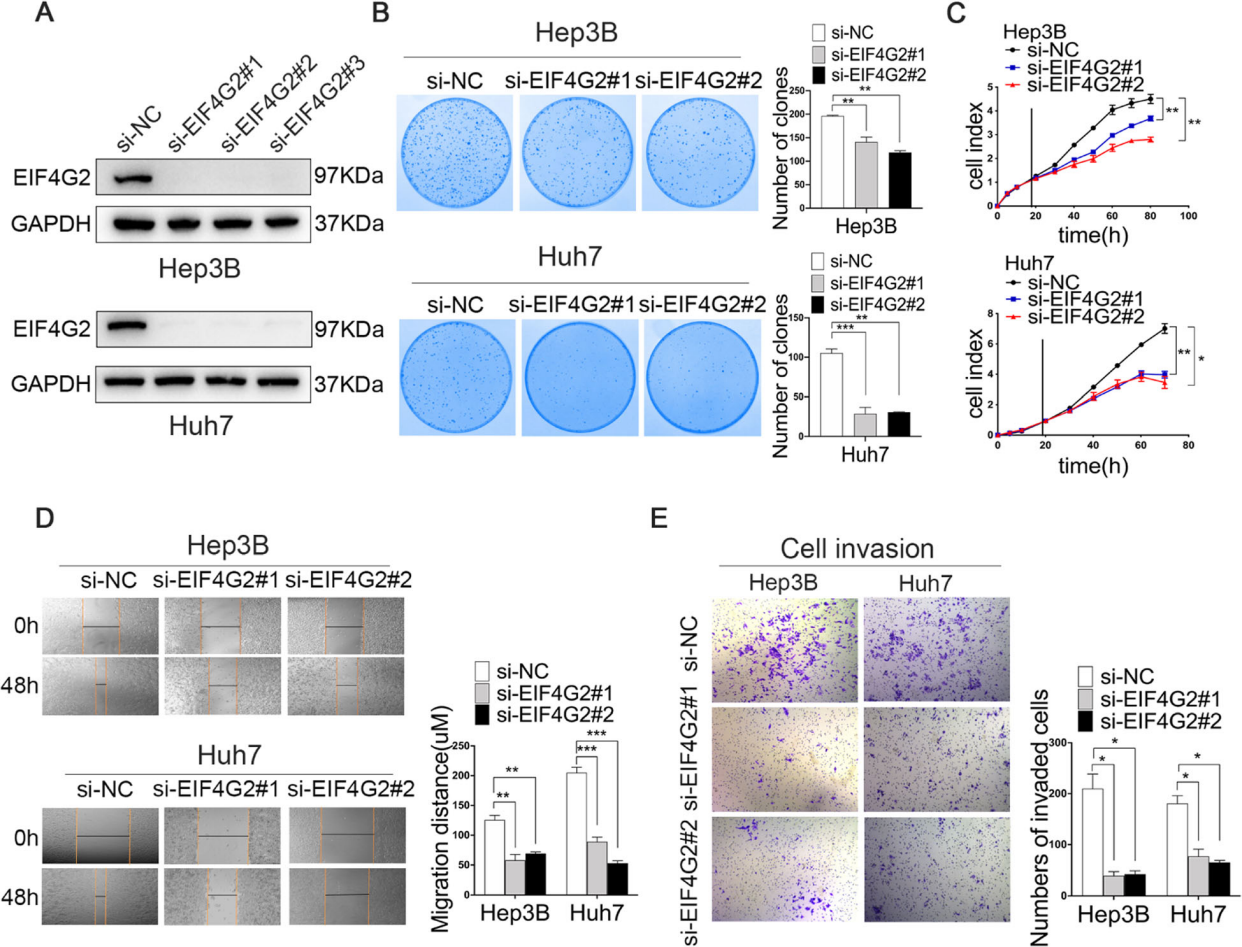
EIF4G2 facilitates HCC growth and metastasis in vitro. **a** HCC cells Hep3B and Huh7 cells were transfected with negative control (NC) or three different si-RNAs of EIF4G2. The knockdown efficiency of EIF4G2 was measured by Western blot. **b** Functions of EIF4G2 knockdown on HCC cells proliferation were performed by colony formation assay. **c** RTCA analysis of cells proliferation in Hep3B and Huh7 cells. **d** Functions of EIF4G2 knockdown on HCC cells migration were determined by scratch experiment. **e** Effects of EIF4G2 knockdown on HCC cells invasion were detected by transwell invasion assays. *p < 0.05, **p < 0.01, ***p < 0.001

Next, we studied the biological functions of EIF4G2 in HCC cells. Three siRNAs targeting EIF4G2 were used to determine the two best knockdown efficiencies by Western blot (Fig. 2a). Colony formation assays and RTCA were conducted to address the effect of EIF4G2 on HCC cell growth. The number of clones of the si-EIF4G2 groups was obviously less than that of the ctrl group (Fig. 2b). Besides, the RTCA results revealed that EIF4G2 knockdown significantly inhibited cell growth in both HCC cell lines (Fig. 2c). On the other hand, the scratch experiment demonstrated that the scratch width narrowed more obviously in the ctrl group (Fig. 2d). Subsequently, the transwell invasion assay was adopted to study cell invasion. The results displayed that the number of invaded cells of si-EIF4G2 groups was less than that in the ctrl group (Fig. 2e). In general, downregulation of EIF4G2 suppressed HCC cell growth and metastasis ability.

### EIF4G2 activates the ERK signaling pathway in HCC

**Fig. 3:**
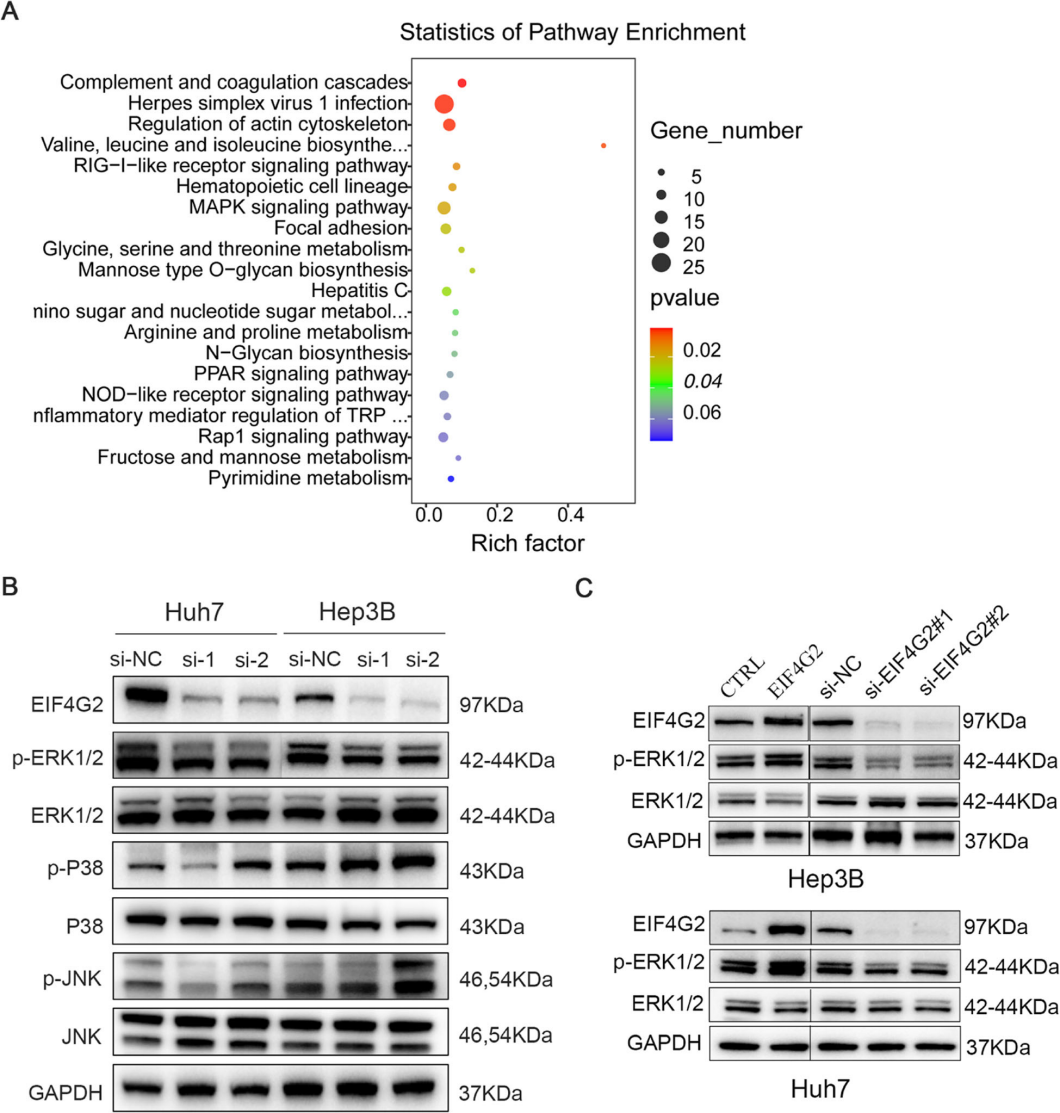
EIF4G2 activates the ERK signaling pathway in HCC. **a** KEGG pathway enrichment analysis of top 20 differentially expressed signaling pathways in EIF4G2 knockdown group compared with ctrl group. **b** MAPK signaling pathway was analyzed by Western blot. **c** ERK signaling pathway was tested by Western blot

To further explore the mechanism of EIF4G2 in HCC, we performed transcriptional profiling of EIF4G2 knockdown Hep3B cells compared with the control group. KEGG pathway enrichment analysis of differentially expressed genes (*p* < 0.05 and absolute value of log2-fold change > 1) revealed that the mitogen-activated protein kinases (MAPK) signaling was one of the top 20 differentially expressed signaling pathways (Fig. 3a). Then, we performed Western blot to verify whether the MAPK signaling pathway was related to the role of EIF4G2 in HCC. In two HCC cell lines, we transfected EIF4G2 siRNA, and then the MAPK signaling pathway, including extracellular-signal-regulated kinases (ERK), P38 MAPK, and c-Jun N-terminal kinases (JNK) signaling, was detected. The results showed that p-ERK 1/2 was obviously suppressed in the two si-EIF4G2 groups versus control group in both two cell lines. While the expression of p-P38 and p-JNK was not consistent in two cell lines or in two si-EIF4G2 groups (Fig. 3b). Thus, ERK signaling was selected for further study. As shown in Fig. 3c, the p-ERK1/2 expression was significantly reduced in the EIF4G2 silencing groups, and overexpression of EIF4G2 improved the level of p-ERK1/2. All these results indicated that EIF4G2 facilitated HCC progression by the ERK signaling pathway. To gain further insight into the role of the ERK signaling pathway in HCC development, we used an inhibitor of ERK (U0126) to explore it. Taken together, inhibition of ERK suppressed HCC development in vitro (Fig. S2A-D). On the other hand, we also found that the mRNA level of Ras protein–specific guanine nucleotide-releasing factor 1 (RASGRF1), Rac family small GTPase 2 (RAC2) and dual specificity phosphatase 3 (DUSP3) was differentially expressed in si-EIF4G2 group compared to the ctrl (Fig. S2E-G), which were reported to regulate the ERK signaling [17, 18].

### EIF4G2 knockdown suppresses tumorigenesis in vivo

**Fig. 4:**
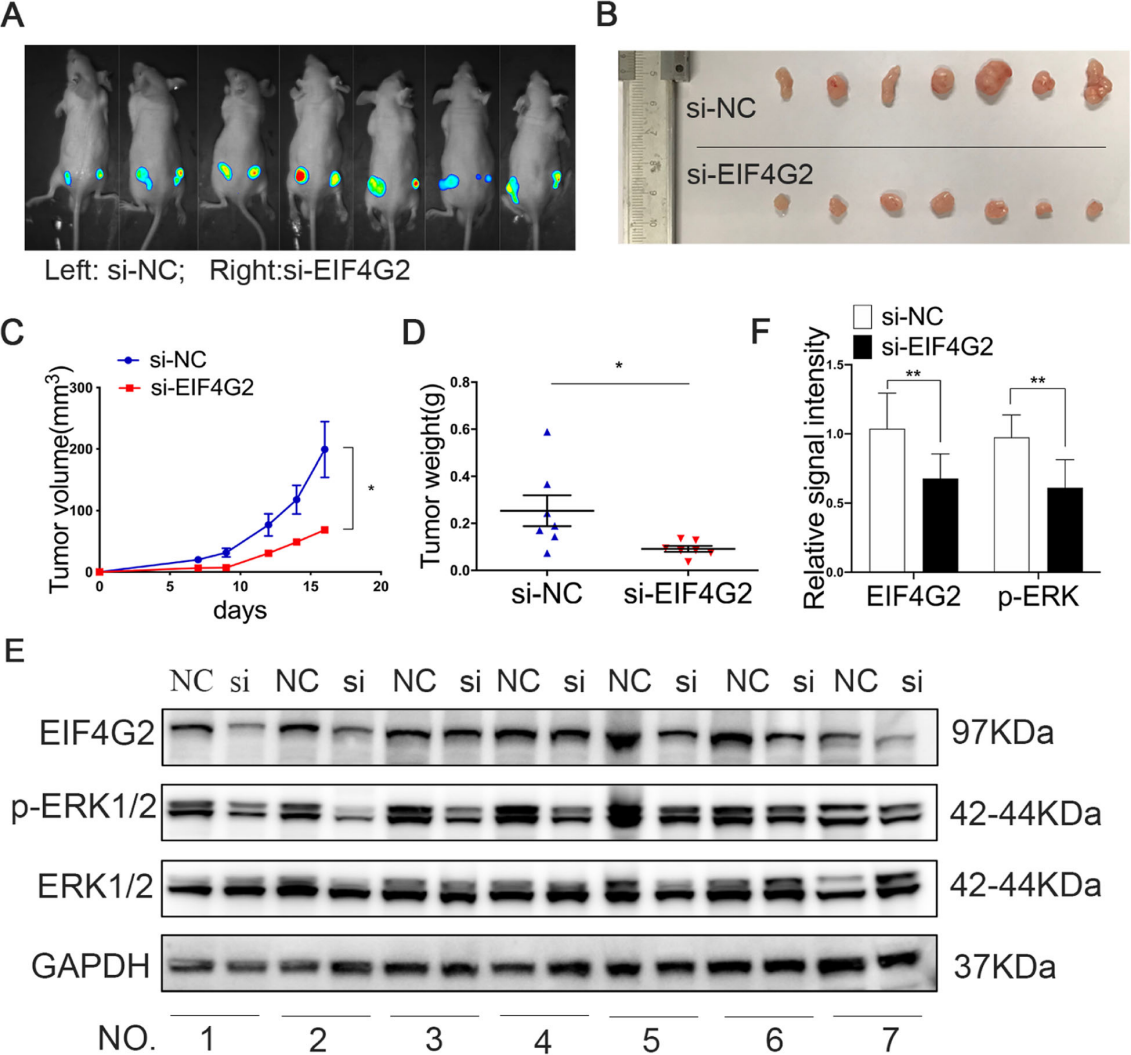
EIF4G2 knockdown suppresses tumorigenesis in vivo. **a** Fluorescent photos of nude mice bearing HCC tumors. **b** Representative of HCC tumors from NC and si-EIF4G2 groups. **c** The tumor growth curves. **d** HCC tumor weight was measured, and the results were presented as mean ± S.D. **e** The expression of EIF4G2, ERK1/2 and p-ERK1/2 proteins in mice tumor were measured with Western blot. **f** Relative signal intensity of EIF4G2 and p-ERK1/2 proteins level of WB. *p < 0.05, ** *p* < 0.01

Equal numbers of Huh7-Luc cells (1 × 10^6^ cells) infected with si-EIF4G2 and NC were subcutaneously injected into the backs of female nude mice. The left side of the mice was the ctrl group, and the right side indicated the experimental group (Fig. 4a). Tumor volumes of the two groups were measured every two days, and a tumor growth curve was drawn. The curves revealed that there was a significant tumor growth inhibition in the EIF4G2 silencing group (Fig. 4c). The mice were sacrificed after 18 days, and the tumor volume and weight of the two groups were determined (Fig. 4b-d). Obviously, downregulation of EIF4G2 suppressed tumor growth in vivo. The expression of EIF4G2 was further confirmed by Western blot. In addition, the levels of the ERK1/2 and p-ERK1/2 were also detected. The level of p-ERK1/2 was lower in the si-EIF4G2 group than in the NC group (Fig. 4e-f).

### EIF4G2 is negatively regulated by miR-144

**Fig. 5:**
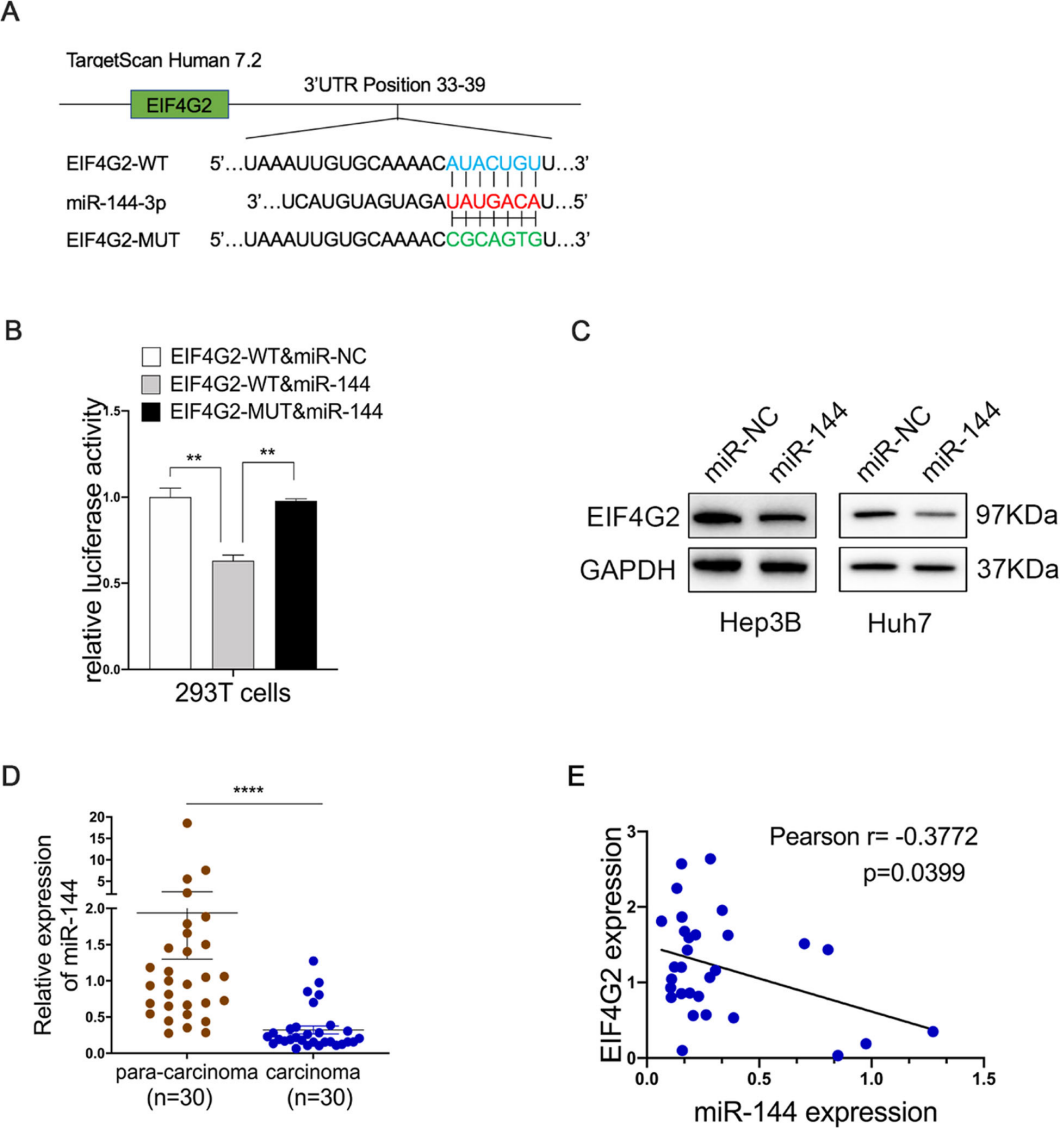
EIF4G2 is negatively regulated by miR-144. **a** Putative binding sites between miR-144 and EIF4G2-WT or EIF4G2-MUT 3’UTR position. **b** Luciferase reporter vectors of EIF4G2-WT or EIF4G2-MUT and miR-144 overexpression vectors were co-transfected into 293 T cells. Relative luciferase activity was assessed 48 h later. (C) Western blot analysis of EIF4G2 level. **d** The expression of miR-144 in HCC tumor tissues and matched para-cancer tissues. **e** Pearson association analysis of the miR-144 level and EIF4G2 protein expression. **p < 0.01, ****p < 0.0001 WT: the wild-type vector, MUT: the mutant vector

Considering the role of miRNAs in cancer development by restraining targeted mRNAs, we used online database websites to predict the possible miRNAs regulating EIF4G2 expression post-transcriptionally. As shown in Fig. 5a, miR-144 might bind with the 3′-UTR of EIF4G2. A luciferase reporter assay exhibited that overexpression of miR-144 inhibited EIF4G2-WT luciferase activity but not EIF4G2-MUT luciferase activity (Fig. 5b). The protein level of EIF4G2 was remarkably repressed after transfecting the miR-144 overexpression vector into Hep3B and Huh7 cells (Fig. 5c). Furthermore, in our 30 paired HCC samples, the expression of miR-144 was significantly lower in HCC tumor tissues than in matched para-cancer tissues (Fig. 5d). Further Pearson correlation analysis demonstrated that the miR-144 expression and EIF4G2 protein levels were negatively correlated in 30 paired HCC samples (Fig. 5e). All of these data confirmed that EIF4G2 could be negatively regulated by miR-144.

### MiR-144 suppresses HCC development in vitro and in vivo

**Fig. 6:**
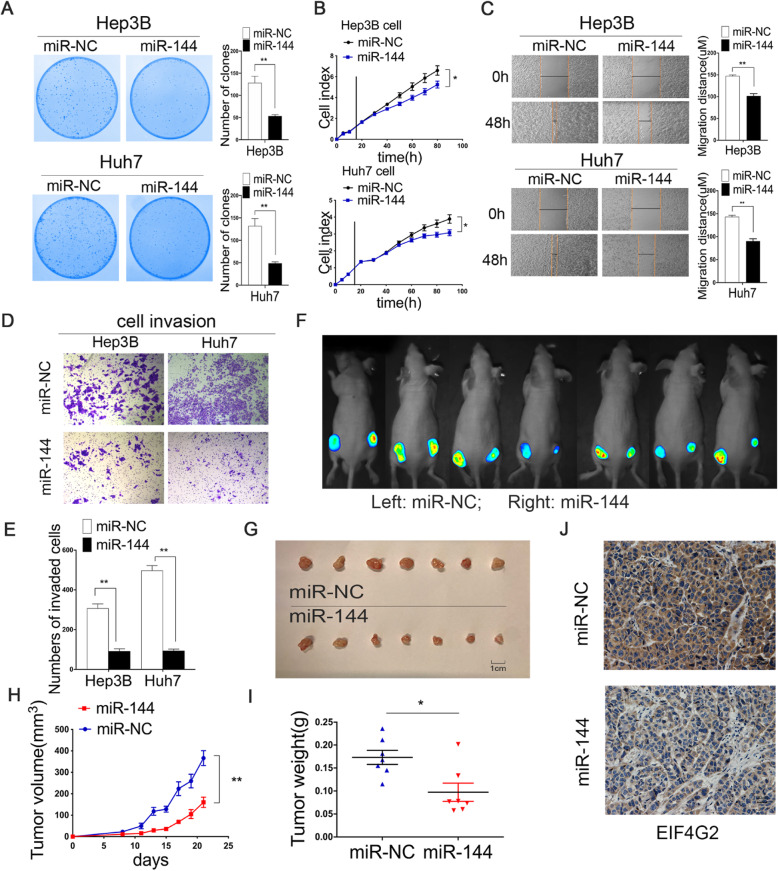
MiR-144 suppresses HCC development in vitro and in vivo. **a** Functions of overexpression of miR-144 on Hep3B and Huh7 cells were determined by colony formation assay. **b** RTCA analysis of cells proliferation in two HCC cells (The vertical line represented the time of cell transfection). **c** Cells migration ability was assessed by wound healing assay. **d** Cells invasion ability was measured by transwell invasion assay. **e** Statistical analysis of the invaded cells. **f** Fluorescent photos of nude mice bearing HCC tumors. **g** Representative of HCC tumors from NC and miR-144 overexpression groups. **h** The tumor growth curves. **i** HCC tumor weight was measured. **j** Representative images of EIF4G2 IHC staining of mice tumor sections from NC and miR-144 group. * *p* < 0.05, **p < 0.01

There have been some studies on the antitumor effect of miR-144 [19–21]. In our study, we performed a series of experiments to explore the effect of miR-144 in HCC. The efficiency of miR-144 overexpression was determined by qRT-PCR (Fig. S1C). The colony formation assays validated that Hep3B and Huh7 cells formed fewer colonies in the miR-144 overexpressed group than in the ctrl group (Fig. 6a). The results of RTCA showed that overexpression of miR-144 could inhibit HCC cellular growth ability (Fig. 6b). Besides, the wound healing and transwell assays demonstrated that upregulation of miR-144 suppressed HCC cell migration (Fig. 6c) and invasion (Fig. 6d-e). In vivo, the equal numbers of Huh7-Luc cells (1 × 10^6^ cells) infected with miR-144 overexpressed lentivirus and control lentivirus vectors were subcutaneously injected into female nude mice (Fig. S1D). The left flank of the mice was the control group, and the right flank was the experimental group. Fluorescent photos of nude mice displayed an obvious inhibitory effect of miR-144 on tumorigenesis (Fig. 6f). The tumor sizes were measured every 2 days (Fig. 6g-h). The mice were sacrificed after 22 days and the tumors were weighed (Fig. 6i). IHC staining results displayed that mouse tumors from the miR-144 overexpression group were remarkably reduced with EIF4G2 expression compared with the NC group (Fig. 6j). These results implied that miR-144 significantly suppressed HCC xenografts in nude mice. In short, our results were consistent with other studies, in which miR-144 played an antitumor role in HCC.

### Functions of EIF4G2 in HCC are regulated by miR-144

**Fig. 7:**
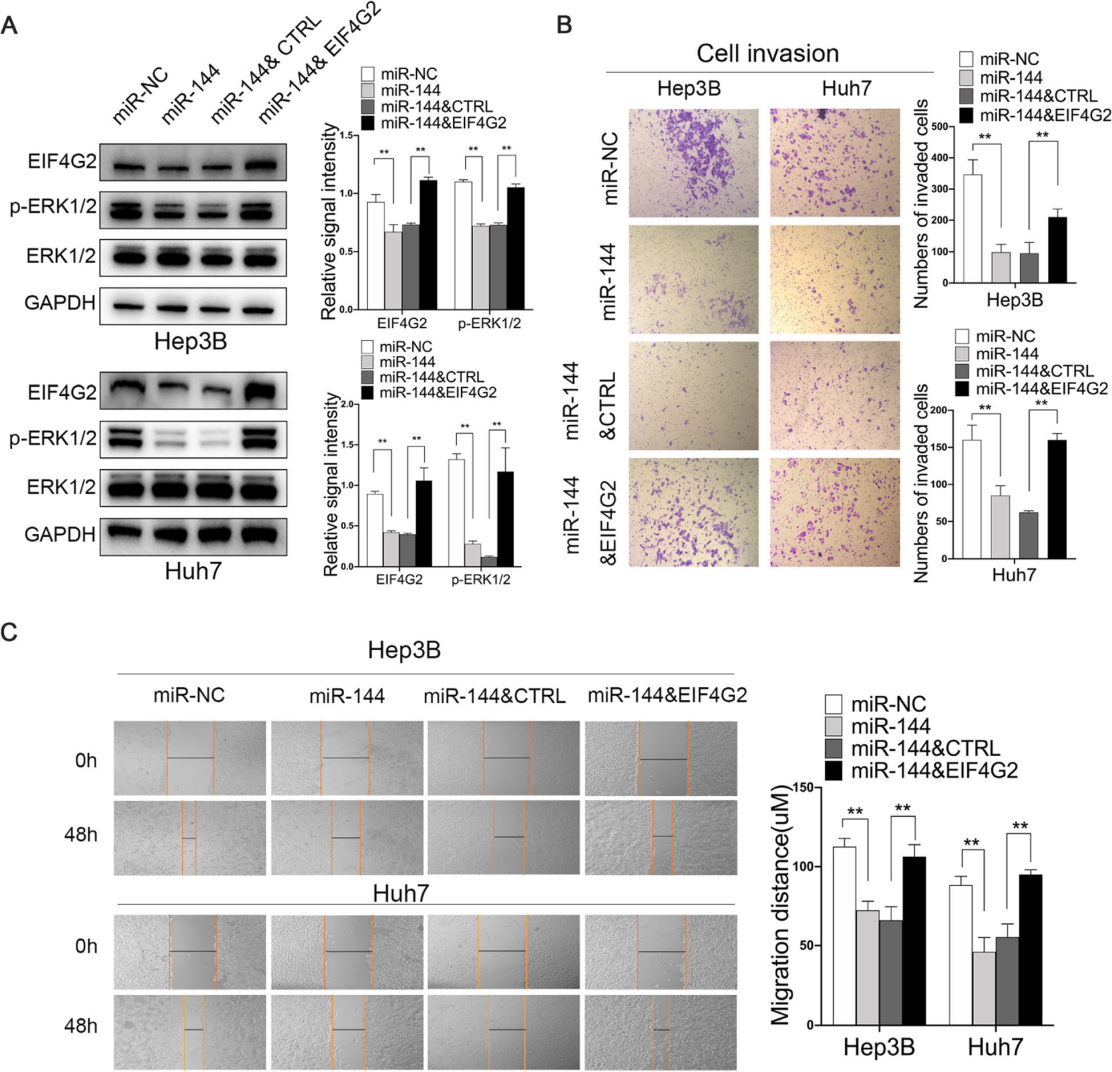
Functions of EIF4G2 in HCC are regulated by miR-144. **a** Western blot analysis of EIF4G2, ERK1/2 and p-ERK1/2 expression. **b** The migration ability was performed with scratch experiments. **c** The invasion ability was assessed by transwell invasion assays. **p < 0.01

Furthermore, we found that the inhibitory effect of miR-144 could be partially reversed by EIF4G2 overexpression. Hep3B and Huh7 cells were transfected with miR-NC, miR-144, miR-144 and ctrl vector, or miR-144 and EIF4G2 overexpression vector. As shown in Fig. 7a, overexpression of EIF4G2 reversed the ERK pathway inhibition by miR-144. Besides, scratch experiments and transwell assays demonstrated that miR-144-induced suppression of HCC cell migration and invasion was remarkably reversed by restoration of EIF4G2 (Fig. 7b-c). The results verified that miR-144 might be the upstream regulatory factor of EIF4G2 and that miR-144 suppressed HCC development by targeting EIF4G2.

## Discussion

HCC is a serious health-threatening disease that has a very poor prognosis due to the lack of effective prognostic and therapeutic targets [22, 23]. In this study, we first showed the evidence that EIF4G2 was an unfavorable prognostic marker in HCC and that suppression of EIF4G2 could inhibit HCC development.

Many studies have suggested that EIF4G2-dependent mRNAs were specifically involved in pro-oncogenic activities, such as cell proliferation, anti-apoptosis, tumor invasion, metastasis and angiogenesis [6, 7, 24–28]. Therefore, EIF4G2 has been found to be associated with tumor development and treatments. Downregulation of EIF4G2 could induce reacquisition of chemosensitivity to paclitaxel in ovarian cancer [29] and enhance cisplatin chemosensitivity in nonsmall cell lung cancer [30]. Here, we first reported that EIF4G2 was abnormally enhanced in HCC tissues and that higher EIF4G2 was associated with some clinicopathological characteristics, such as T stage, TNM stage, recurrence, and poorer DFS of HCC patients. The OS results in our study were not significantly different, which might be because the number of our samples was too small (*n* = 89) compared with the database (*n* = 365). Further study revealed that EIF4G2 showed no difference between HBV+ HCC cases and HBV- HCC cases, demonstrating that EIF4G2 was not associated with HBV (Fig. S1F). Besides, the positive correlation between EIF4G2 and PDL1 expression reflected that they might be jointly involved in the disease progression and immune escape of HCC, which deserves further study. Moreover, EIF4G2 strongly promoted HCC cell growth and metastasis and promoted HCC xenografts in nude mice. Mechanically, in our study, the results of transcriptional profiling demonstrated that EIF4G2 knockdown could obviously inhibit the MAPK signaling pathway. MAPKs in mammals mainly include three major subfamilies: JNK, p38 MAPK and ERK [31, 32]. Here, we tested all three signaling pathways by Western blot assay to find out which one was implicated. As shown in Fig. 3b, both si-EIF4G2 groups caused p-ERK suppression in Huh7 and Hep3B cells. In contrast, p-p38 and p-JNK showed a higher expression in the si-EIF4G2 groups of Hep3B cells and showed the opposite trend in si-EIF4G2#1 versus si-EIF4G2#2 in Huh7 cells. Our data were consistent with other studies, in which ERK signaling was shown to be one of the most important pathways in cell proliferation and metastasis, and activation of the ERK signaling pathway is closely related to HCC prognosis and development, and is detectable in nearly half of early HCC patients and almost all patients with advanced HCC [33, 34]. And the p38 MAPK and JNK signaling pathways play a role in cell apoptosis and proinflammatory responses [35, 36]. Taken together, we speculated that EIF4G2 promoted HCC development through the ERK signaling pathway. Consistent with this, a study revealed that deletion of EIF4G2 caused decreased expression of MAP3K3 and SOS1, which were reported to be upstream of the ERK/MAPK signaling pathway [37, 38]. While, in our study, there was no significant difference of MAP3K3 and SOS1, this might be because the cell lines used were different. On the other hand, we found the mRNA level of RASGRF1 and RAC2 were suppressed and the level of DUSP3 was increased in the downregulation of EIF4G2 according to the result of transcriptional profiling. These three molecules were all involved in the ERK/MAPK pathway. RASGRF1, like the SOS1 (mentioned above), is one of the Ras-specific guanine nucleotide exchange factors, which can convert Ras from inactive GDP-Ras form to GTP-Ras activate form, then activating the downstream ERK signaling [39]. RAC2 is one of Rac GTPase, which can activate the Raf together with the Cdc42hs and RhoA, and then increasing ERK1/2 activity [40]. While DUSP3 is one of the dual specific protein phosphatases, which can dephosphorylate and inactivate ERK1/2 [41]. These data further suggested that knockdown of EIF4G2 inhibit the ERK pathway by regulating the upstream genes of the ERK/MAPK signaling pathway.

Analysis of GEPIA data showed that the level of EIF4G2 mRNA was not significantly different between HCC tissues and paracarcinoma tissues (Fig. S1E), while the protein level of EIF4G2 was obviously different. The results reminded us of the regulatory mechanism of miRNAs. miRNAs participate in posttranscriptional regulation by binding to the 3’UTR of targeted mRNAs, preventing protein translation. Using some website databases containing TargetScan, miRCODE and starBase, we found that miR-144 was likely a new regulator to modulate EIF4G2 because there were several conserved binding sites between them. miR-144, a well-known tumor suppressor, has been found to be involved in the development of several cancers, including HCC. A comprehensive analysis of miR-144 in HCC revealed that many genes and signaling pathways were regulated by miR-144, including the Toll-like receptor pathway, p53 signaling pathway, and cell cycle-associated proteins [42]. In addition, miR-144 could inhibit human hepatocellular carcinoma by regulating CCNB1 [43], repress the mTOR-VEGF pathway by targeting SGK3 in HCC [44], and reverse the chemoresistance of HCC cells by suppressing Nrf2 [45]. In our work, EIF4G2 was found to be negatively regulated by miR-144, and the levels of miR-144 and EIF4G2 were inversely associated with each other in HCC samples. Consistent with other studies, we demonstrated that miR-144 suppressed HCC development in vitro and in vivo. Moreover, we discovered that re-expression of EIF4G2 could attenuate the inhibitory action of miR-144 on HCC.

## Conclusion

Our discoveries first revealed that EIF4G2 was increased in HCC tissues, and upregulation of EIF4G2 was strongly associated with a poorer prognosis of HCC patients. In vitro and in vivo assays demonstrated that downregulation of EIF4G2 could inhibit HCC cell growth, metastasis and tumorigenesis through suppression of the ERK signaling pathway. Finally, we found that miR-144, as a tumor suppressor, could negatively regulate EIF4G2 expression. Our findings revealed that EIF4G2 might be a promising therapeutic target in HCC that deserves further study in the future. And we may be able to establish a screening system, adding this molecule to the pathological histochemistry of HCC patients to predict the prognosis of liver cancer patients.

## Supplementary Information

**Additional file 1: Figure S1.** Supplementary data. **Figure S2.** Inhibition of ERK suppresses HCC growth and metastasis in vitro. **Table S1.** Clinical HCC patients’ information. **Table S2.** Sequences of primers. **Table S3.** Sequences of siRNAs.

## Data Availability

The datasets analyzed during the current study are available from the corresponding author on reasonable request.

## Abbreviations

HCC: Hepatocellular carcinoma
TMAs: Tissue microarrays
IHC: Immunohistochemistry
miRNAs: MicroRNAs
TACE: Transcatheter arterial chemoembolization
eIFs: Eukaryotic translation initiation factors
EIF4G2: Eukaryotic translation initiation factor 4 gamma 2
Huh7-Luc: Huh7 cells stably expressing luciferase
RTCA: Real-Time Cell Analysis
RT-qPCR: Quantitative real-time polymerase chain reaction
WT: Wild-type
MUT: Mutant
OS: Overall survival
DFS: Disease-free survival
ERK: Extracellular-signal-regulated kinases
JNK: C-Jun N-terminal kinases
RASGRF1: Ras protein–specific guanine nucleotide-releasing factor 1
RAC2: Rac family small GTPase 2
DUSP3: Dual specificity phosphatase 3

## References

[1] Siegel RL, Miller KD, Jemal A (2018). Cancer statistics, 2018. CA Cancer J Clin.

[2] Bruix J, Reig M, Sherman M (2016). Evidence-based diagnosis, staging, and treatment of patients with hepatocellular carcinoma. Gastroenterology..

[3] Bhat M, Robichaud N, Hulea L, Sonenberg N, Pelletier J, Topisirovic I (2015). Targeting the translation machinery in cancer. Nat Rev Drug Discov.

[4] Gantenbein N, Bernhart E, Anders I, Golob-Schwarzl N, Krassnig S, Wodlej C (2018). Influence of eukaryotic translation initiation factor 6 on non-small cell lung cancer development and progression. Eur J Cancer.

[5] Golob-Schwarzl N, Krassnig S, Toeglhofer AM, Park YN, Gogg-Kamerer M, Vierlinger K (2017). New liver cancer biomarkers: PI3K/AKT/mTOR pathway members and eukaryotic translation initiation factors. Eur J Cancer.

[6] Lewis SM, Cerquozzi S, Graber TE, Ungureanu NH, Andrews M, Holcik M (2007). The eIF4G homolog DAP5/p97 supports the translation of select mRNAs during endoplasmic reticulum stress. Nucleic Acids Res.

[7] Warnakulasuriyarachchi D, Cerquozzi S, Cheung HH, Holcik M (2004). Translational induction of the inhibitor of apoptosis protein HIAP2 during endoplasmic reticulum stress attenuates cell death and is mediated via an inducible internal ribosome entry site element. J Biol Chem.

[8] Nousch M, Reed V, Bryson-Richardson RJ, Currie PD, Preiss T (2007). The eIF4G-homolog p97 can activate translation independent of caspase cleavage. RNA..

[9] Emmrich S, Engeland F, El-Khatib M, Henke K, Obulkasim A, Schoning J (2016). miR-139-5p controls translation in myeloid leukemia through EIF4G2. Oncogene..

[10] Mazan-Mamczarz K, Zhao XF, Dai B, Steinhardt JJ, Peroutka RJ, Berk KL (2014). Down-regulation of eIF4GII by miR-520c-3p represses diffuse large B cell lymphoma development. PLoS Genet.

[11] Xie X, Li YS, Xiao WF, Deng ZH, He HB, Liu Q (2017). MicroRNA-379 inhibits the proliferation, migration and invasion of human osteosarcoma cells by targetting EIF4G2. Biosci Rep.

[12] The Human Protein Atlas Accessed 7 July 2020 [Available from: https://www.proteinatlas.org/ENSG00000110321-EIF4G2/pathology/liver+cancer - Intensity.

[13] GEPIA [Available from: http://gepia.cancer-pku.cn/detail.php?gene=EIF4G2. Accessed 7 June 2019.

[14] Bartel DP (2009). MicroRNAs: target recognition and regulatory functions. Cell..

[15] Lou G, Chen L, Xia C, Wang W, Qi J, Li A, et al. MiR-199a-modified exosomes from adipose tissue-derived mesenchymal stem cells improve hepatocellular carcinoma chemosensitivity through mTOR pathway. J Exp Clin Cancer Res. 2020;39(1):4. 10.1186/s13046-019-1512-5 PMC6941283 31898515

[16] Liu Y, Lou G, Norton JT, Wang C, Kandela I, Tang S (2017). 6-Methoxyethylamino-numonafide inhibits hepatocellular carcinoma xenograft growth as a single agent and in combination with sorafenib. FASEB J.

[17] Zhou B, Wang ZX, Zhao Y, Brautigan DL, Zhang ZY (2002). The specificity of extracellular signal-regulated kinase 2 dephosphorylation by protein phosphatases. J Biol Chem.

[18] Gavino C, Hamel N, Zeng JB, Legault C, Guiot MC, Chankowsky J, et al. Impaired RASGRF1/ERK-mediated GM-CSF response characterizes CARD9 deficiency in French-Canadians. J Allergy Clin Immunol 2016;137(4):1178–1188 e7. 10.1016/j.jaci.2015.09.016 26521038

[19] Lan F, Yu H, Hu M, Xia T, Yue X (2015). miR-144-3p exerts anti-tumor effects in glioblastoma by targeting c-met. J Neurochem.

[20] Jahanbani I, Al-Abdallah A, Ali RH, Al-Brahim N, Mojiminiyi O (2018). Discriminatory miRNAs for the Management of Papillary Thyroid Carcinoma and Noninvasive Follicular Thyroid Neoplasms with papillary-like nuclear features. Thyroid..

[21] Wang H, Wang A, Hu Z, Xu X, Liu Z, Wang Z (2016). A critical role of miR-144 in diffuse large B-cell lymphoma proliferation and invasion. Cancer Immunol Res.

[22] Nakagawa S, Wei L, Song WM, Higashi T, Ghoshal S, Kim RS (2016). Molecular liver Cancer prevention in cirrhosis by organ Transcriptome analysis and Lysophosphatidic acid pathway inhibition. Cancer Cell.

[23] Goossens N, Sun X, Hoshida Y (2015). Molecular classification of hepatocellular carcinoma: potential therapeutic implications. Hepat Oncol.

[24] Weingarten-Gabbay S, Khan D, Liberman N, Yoffe Y, Bialik S, Das S (2014). The translation initiation factor DAP5 promotes IRES-driven translation of p53 mRNA. Oncogene..

[25] de la Parra C, Ernlund A, Alard A, Ruggles K, Ueberheide B, Schneider RJ (2018). A widespread alternate form of cap-dependent mRNA translation initiation. Nat Commun.

[26] Bellsolell L, Cho-Park PF, Poulin F, Sonenberg N, Burley SK (2006). Two structurally atypical HEAT domains in the C-terminal portion of human eIF4G support binding to eIF4A and Mnk1. Structure..

[27] Marash L, Liberman N, Henis-Korenblit S, Sivan G, Reem E, Elroy-Stein O (2008). DAP5 promotes cap-independent translation of Bcl-2 and CDK1 to facilitate cell survival during mitosis. Mol Cell.

[28] Hundsdoerfer P, Thoma C, Hentze MW (2005). Eukaryotic translation initiation factor 4GI and p97 promote cellular internal ribosome entry sequence-driven translation. Proc Natl Acad Sci U S A.

[29] Zhao H, Wang A, Zhang Z. LncRNA SDHAP1 confers paclitaxel resistance of ovarian cancer by regulating EIF4G2 expression via miR-4465. J Biochem. 2020. 10.1093/jb/mvaa036 32211849

[30] Hao GJ, Hao HJ, Ding YH, Wen H, Li XF, Wang QR (2017). Suppression of EIF4G2 by miR-379 potentiates the cisplatin chemosensitivity in nonsmall cell lung cancer cells. FEBS Lett.

[31] Dong C, Davis RJ, Flavell RA (2002). MAP kinases in the immune response. Annu Rev Immunol.

[32] Hommes DW, Peppelenbosch MP, van Deventer SJ (2003). Mitogen activated protein (MAP) kinase signal transduction pathways and novel anti-inflammatory targets. Gut..

[33] Liu Y, Zhang X, Yang B, Zhuang H, Guo H, Wei W (2018). Demethylation-induced overexpression of Shc3 drives c-Raf-independent activation of MEK/ERK in HCC. Cancer Res.

[34] Ito Y, Sasaki Y, Horimoto M, Wada S, Tanaka Y, Kasahara A (1998). Activation of mitogen-activated protein kinases/extracellular signal-regulated kinases in human hepatocellular carcinoma. Hepatology..

[35] Arthur JS, Ley SC (2013). Mitogen-activated protein kinases in innate immunity. Nat Rev Immunol.

[36] Sabio G, Davis RJ (2014). TNF and MAP kinase signalling pathways. Semin Immunol.

[37] Sugiyama H, Takahashi K, Yamamoto T, Iwasaki M, Narita M, Nakamura M (2017). Nat1 promotes translation of specific proteins that induce differentiation of mouse embryonic stem cells. Proc Natl Acad Sci U S A.

[38] Craig EA, Stevens MV, Vaillancourt RR, Camenisch TD (2008). MAP 3Ks as central regulators of cell fate during development. Dev Dyn.

[39] Lowy DR, Willumsen BM (1993). Function and regulation of ras. Annu Rev Biochem.

[40] Frost JA, Xu S, Hutchison MR, Marcus S, Cobb MH (1996). Actions of rho family small G proteins and p21-activated protein kinases on mitogen-activated protein kinase family members. Mol Cell Biol.

[41] Alonso A, Saxena M, Williams S, Mustelin T (2001). Inhibitory role for dual specificity phosphatase VHR in T cell antigen receptor and CD28-induced Erk and Jnk activation. J Biol Chem.

[42] Liang HW, Ye ZH, Yin SY, Mo WJ, Wang HL, Zhao JC (2017). A comprehensive insight into the clinicopathologic significance of miR-144-3p in hepatocellular carcinoma. Onco Targets Ther.

[43] Gu J, Liu X, Li J, He Y (2019). MicroRNA-144 inhibits cell proliferation, migration and invasion in human hepatocellular carcinoma by targeting CCNB1. Cancer Cell Int.

[44] Wu M, Huang C, Huang X, Liang R, Feng Y, Luo X (2017). MicroRNA-144-3p suppresses tumor growth and angiogenesis by targeting SGK3 in hepatocellular carcinoma. Oncol Rep.

[45] Zhou S, Ye W, Zhang Y, Yu D, Shao Q, Liang J (2016). miR-144 reverses chemoresistance of hepatocellular carcinoma cell lines by targeting Nrf2-dependent antioxidant pathway. Am J Transl Res.

