# Supplementary material for: MiR-144-3p-mediated dysregulation of EIF4G2 contributes to the development of hepatocellular carcinoma through the ERK pathway
Source: J Exp Clin Cancer Res. 2021 Feb 1;40:53. doi: 10.1186/s13046-021-01853-6 (PMC7852102; doi:10.1186/s13046-021-01853-6)
Supplement: Supplementary file 1 — Additional file 1: Figure S1. Supplementary data. Figure S2. Inhibition of ERK suppresses HCC growth and metastasis in vitro. Table S1. Clinical HCC patients’ information. Table S2. Sequences of primers. Table S3. Sequences of siRNAs. [file 13046_2021_1853_MOESM1_ESM.docx]

**
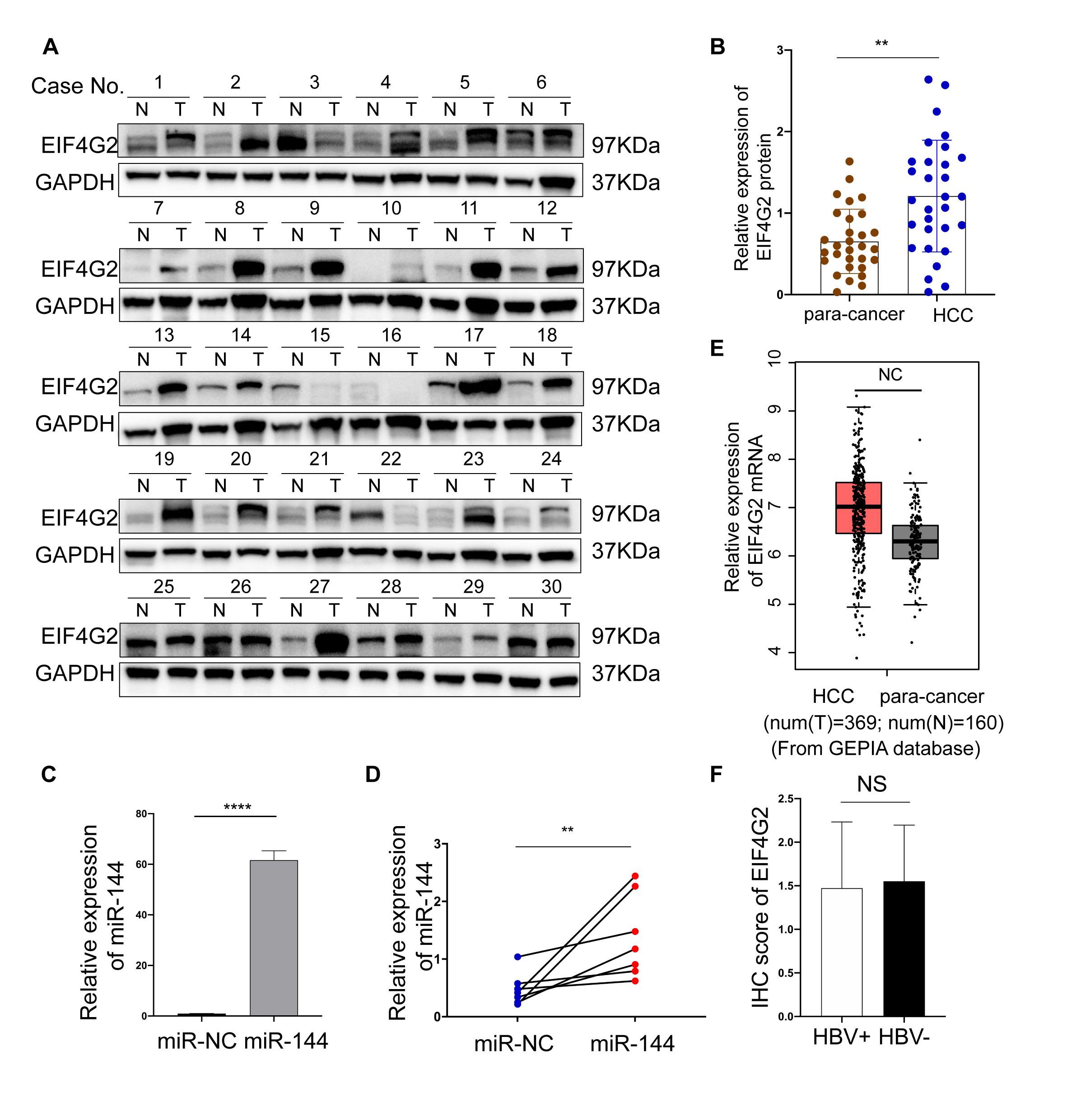
**

**Figure S1** Supplementary data. (A) WB analysis of EIF4G2 level in the 30 paired HCC tissues. (B) Statistical analysis of EIF4G2 expression in 30 paired HCC tissues. (C) The overexpression efficiency of miR-144 was measured by qRT-PCR. (D) qPCR analysis of the miR-144 expression of two groups from 7 mice tumor. (E) EIF4G2 mRNA level in HCC and para-cancer from GEPIA database. (F) EIF4G2 expression in HBV+ and HBV- HCC cases. ***p* < 0.01, *****p* < 0.0001.


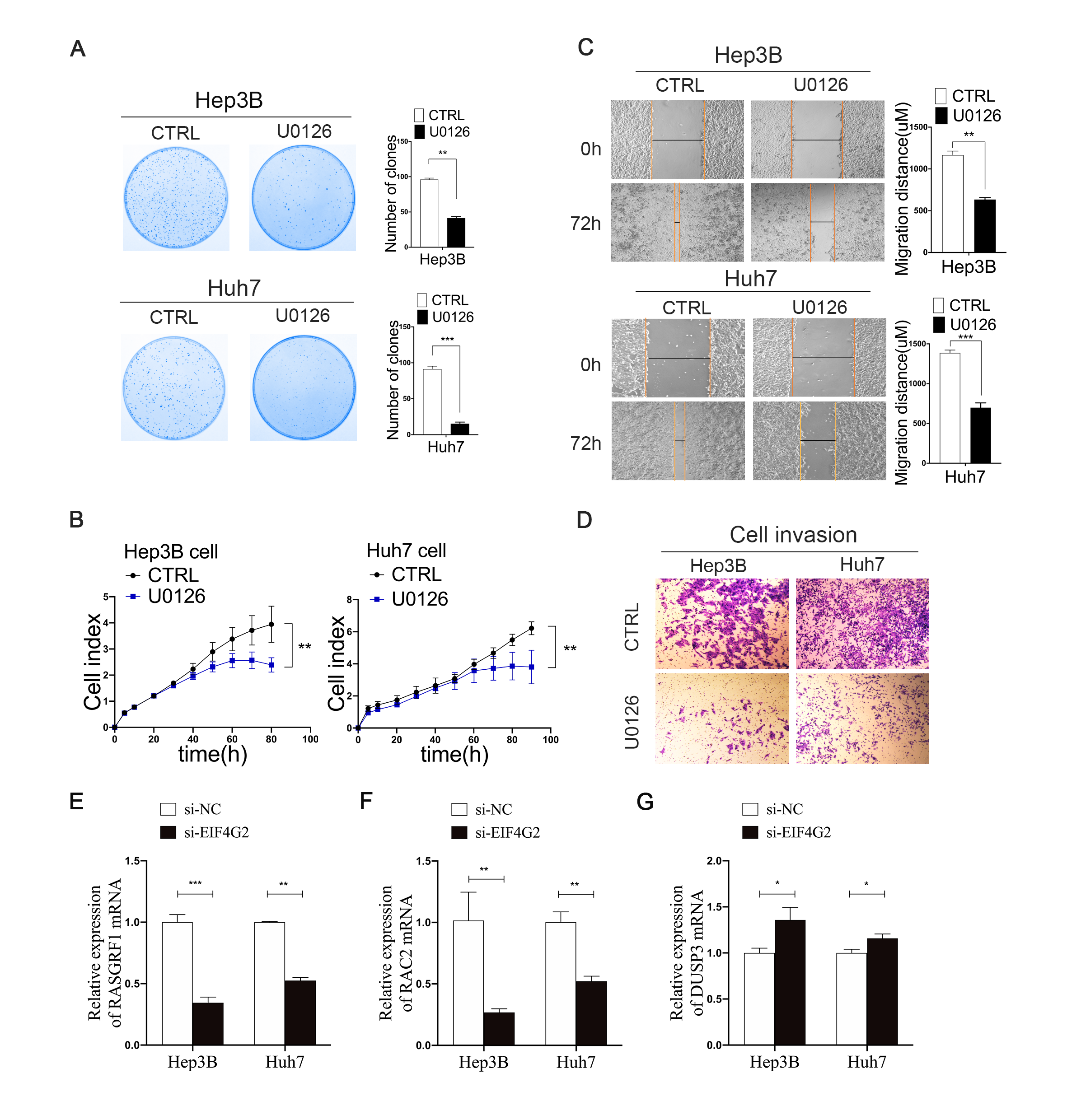


**Figure S2** Inhibition of ERK suppresses HCC growth and metastasis in vitro. (A) Functions of ERK inhibition on HCC cells proliferation were performed by colony formation assay. (B) RTCA analysis of cells proliferation in Hep3B and Huh7 cells. (C) The migration ability of HCC cells was determined by scratch experiment. (D) The invasion ability of cells was detected by transwell invasion assays. (E-G) The mRNA level of RASGRF1, RAC2, DUSP3 was assessed by RT-qPCR. **p* < 0.05, ***p* < 0.01, ****p* < 0.001.

**Table S1** Clinical HCC patients’ information.

| Clinical factor | No. | Clinical factor | No. |
| --- | --- | --- | --- |
| Age (years) |  | Number of lesions |  |
| < 60 | 18 | 1 | 17 |
| ≥60 | 12 | ≥1 | 13 |
| Gender |  | Vessel cancerous embolus |  |
| Male | 20 | Negative | 14 |
| Female | 10 | Positive | 16 |
| HBV infection |  | TNM stage |  |
| YES | 23 | Stage I + stage II | 16 |
| No | 7 | Stage III + stage IV | 14 |
| Liver cirrhosis |  | Child-Pugh stage |  |
| YES | 24 | A | 21 |
| No | 6 | B or C | 9 |
| Tumor size (cm) |  | BCLC^*^ stage |  |
| >5 | 25 | A or B | 20 |
| ≤5 | 5 | C or D | 10 |

BCLC^*^: Barcelona Clinic Liver Cancer

**Table S2** Sequences of primers.

| **Gene** | **Primers** **sequences** |
| --- | --- |
| GAPDH | F: 5’-AGGGCAAAACGCTCAGAAATG-3’ |
|  | R: 5’-GGCTGTTGTCATACTTCTCATGG-3’ |
| EIF4G2 | F: 5’-AGGGCAAAACGCTCAGAAATG-3’ |
| RASGRF  RAC2  DUSP3 | R: 5’-TCCTGAAGATTGCATCATGTCG-3’  F: 5’-TACTCGGCCATGTCACCCTT-3’  R: 5’-GGGTCGTATCGCCCTCATC-3’  F: 5’-CAACGCCTTTCCCGGAGAG-3’  R: 5’-TCCGTCTGTGGATAGGAGAGC-3’  F: 5’-AAGGACTCCGGCATCACATAC-3’  R: 5’-AAGCCTGGTCAATGAAGTCGG-3’ |

**Table S3** Sequences of siRNAs.

| **Gene** | **siRNA sequences** |
| --- | --- |
| si-EIF4G2#1 | Sense: 5’-GCAAUCUUCAGGAAAGUAATT-3′, |
|  | Antisense: 5’-UUACUUUCCUGAAGAUUGCTT-3′ |
| si-EIF4G2#2 | Sense: 5’-GCACUAGACGAGAUGACAATT-3′ |
|  | Antisense: 5’-UUGUCAUCUCGUCUAGUGCTT-3′ |
| si-EIF4G2#3 | Sense: 5’-GCUUCUCGUUUCAGUGCUUTT-3′ |
|  | Antisense: 5’-AAGCACUGAAACGAGAAGCTT-3′ |
| Negative control(NC) | Sense: 5’-UUCUCCGAACGUGUCACGUTT-3′ |
|  | Antisense: 5’-ACGUGACACGUUCGGAGAATT-3′ |
